# Somatic and germline genetic testing pathways in haematological malignancies: Best practice consensus guidelines from the 2025 national meeting organised by UK Cancer Genetics Group (UKCGG), CanGene‐CanVar and the NHS England Haematological Oncology Working Group

**DOI:** 10.1111/bjh.70404

**Published:** 2026-03-03

**Authors:** B. Speight, A. Hamblin, P. Talley, O. Tsoulaki, R. Robinson, P. Dean, J. Khorashad, A. Kulasekararaj, A. L. Godfrey, A. J. Mead, T. P. McVeigh, K. Snape, Consensus Meeting Attendees

**Affiliations:** ^1^ East Anglian Medical Genetics Service, Cambridge Biomedical Campus, Cambridge University Hospitals NHS Foundation Trust Cambridge UK; ^2^ Oxford University Hospitals NHS Foundation Trust & Central & South Genomic Laboratory Hub Oxford UK; ^3^ HMDS, Leeds Teaching Hospitals Trust, St James's University Hospital Leeds UK; ^4^ Genomics Unit, NHS UK London UK; ^5^ Manchester Centre for Genomic Medicine, St Mary's Hospital Manchester UK; ^6^ North East and Yorkshire Genomics Laboratory Hub, St James's University Hospital Leeds UK; ^7^ The Royal Marsden NHS Foundation Trust Sutton UK; ^8^ King's College Hospital NHS Foundation Trust London UK; ^9^ National Institute for Health and Care Research and Wellcome King's Research Facility London UK; ^10^ King's College London London UK; ^11^ Haematopathology & Oncology Diagnostics Service Cambridge University Hospitals NHS Foundation Trust Cambridge UK; ^12^ Medical Research Council (MRC) Weatherall Institute of Molecular Medicine (WIMM) and NIHR Biomedical Research Centre, University of Oxford Oxford UK; ^13^ Institute of Cancer Research London UK; ^14^ South West Thames Regional Genetics Service St George's University Hospitals NHS Foundation Trust London UK

**Keywords:** best practice guidelines, *DDX41*, genetic testing, haematological malignancies, myeloproliferative neoplasms, *TP53*

## Abstract

Genomic technologies including next‐generation sequencing (NGS) and arrays for cytogenetic anomalies are now standard of care in England for the diagnostic evaluation of patients with suspected haematological malignancies. Challenges remain in the management of potential germline findings as a result of NGS panels and copy number variant analyses in haemato‐oncology pathways. The first national consensus meeting in April 2022 organised by the UK Cancer Genetics Group (UKCGGG), in collaboration with the CanGene‐CanVar research programme and the NHS England haemato‐oncology working group led to published best practice recommendations on laboratory and clinical pathways where there was potential to identify germline predisposition to haematological malignancies. On 3 and 4 April 2025, a second national meeting was held to address further challenges in these pathways and review updates in the national landscape subsequent to the 2022 recommendations. The meeting specifically focussed on *TP53*, *DDX41*, myeloproliferative neoplasm driver genes, non‐single nucleotide variation and identification of gene carriers for addition to the National Inherited Cancer Predisposition Register (NICPR) through the National Disease Registration Service (NDRS). Using the format of a pre‐workshop survey followed by structured discussion and in‐meeting polling, high‐level consensus was achieved for UK best practice across these areas.

## BACKGROUND

Genomic technologies, including whole genome sequencing (WGS), large somatic gene panels and cytogenetic analyses in haematological malignancies, are standard of care within the UK National Health Service (NHS). Next‐generation sequencing (NGS) to identify genetic variants is routinely used to inform the diagnosis, prognosis and therapy in patients with a suspected or confirmed haematological malignancy. This area of healthcare continues to present challenges around how to manage and standardise key aspects of the laboratory and clinical pathways for better patient care.

In April 2022, the UK Cancer Genetics Group (UKCGG) in collaboration with the CanGene‐CanVar research programme and the NHS England Haematological Oncology working group organised a first national consensus meeting bringing together Haematologists, Clinical Geneticists, Genetic Counsellors, policy makers, patient representatives and Clinical Scientists. The meeting aimed to achieve consensus across the United Kingdom to standardise aspects of the laboratory and clinical pathways where there was potential to identify a germline predisposition to haematological malignancies. A high level of consensus was achieved, especially on issues regarding standardised reporting, sample types, variant classification, patient support and multidisciplinary team working.^1^ Subsequently, a separate dedicated workshop in collaboration with British Society of Blood and Marrow Transplantation was organised to focus on donor selection for those patients requiring allogenic transplant when potential related donors carry/are at risk of inheriting a germline (likely) pathogenic variant (GPV) predisposing to haematological malignancy.^2^

In April 2025, the UK Cancer Genetics Group (UKCGG) in collaboration with the CanGene‐CanVar research programme and the NHS England Haematological Oncology working group organised a second national consensus meeting to focus on new and remaining challenges in these pathways. This brought together Haematologists, Clinical Geneticists, Genetic Counsellors, policy makers, patient representatives and Clinical Scientists from different areas of the United Kingdom. The purpose of this second follow‐up meeting was to further standardise and improve care pathways, with a focus on *TP53*, *DDX41*, myeloproliferative neoplasm (MPN) driver genes and copy number variants (CNVs)/non‐single nucleotide variants (SNVs). It also aimed to address the identification of individuals with a germline cancer susceptibility gene for the National Inherited Cancer Predisposition Register (NICPR) held by the National Disease Registration Service (NDRS).^3^ High consensus was achieved on issues regarding variant types and clinical scenarios that warrant germline assessment. These best practice recommendations are intended to support decisions around germline assessment and targeted germline testing, and indications for referral to Clinical Genetics for discussion and potential family follow‐up.

### Clinical utility

Decisions regarding whether germline genetic testing is recommended consider the clinical utility balanced against the burden of work associated with variant interpretation, reporting and onward management of the patient and their at‐risk relatives. The clinical utility of a germline genetic test result hinges on the lifetime risk (penetrance) associated with a particular genetic trait, the severity of the associated phenotype and the availability of interventions that can mitigate and/or manage this risk.

It is generally accepted in NHS practice that germline genetic testing should only be offered when all the following circumstances are met^4^:

1. Diagnostic yield of testing is at least 10%.
2. Variants in the gene in question are associated with a serious health condition.
3. The lifetime cancer risk associated with GPV in the gene in question is at least twofold that of the background population (contextualised to the absolute risk of the cancer type in the general population).
4. Burden of work for laboratory and clinical services should be proportionate to the clinical utility of variant identification.

For recessive conditions, testing of partners of carriers of recessive traits should be offered to inform reproductive decision‐making if the carrier frequency of variants in the gene is at least 1 in 70.^5^

In the context of haematological malignancies, targeted germline genetic testing to distinguish between (possibly incidental) variants of germline origin and somatic drivers may have utility in terms of disease prognostication and management, even if clinical utility of identifying presymptomatic carriers is limited.

Specific considerations are required regarding patients with haematological malignancies requiring bone marrow/stem cell transplant, where germline genetic testing of suspicious variants on somatic testing not reaching the threshold for classification as likely pathogenic may be cautiously considered in order to inform donor selection.^2^

### Follow‐up testing for variants of putative germline origin picked up through somatic testing

The European Society for Medical Oncology (ESMO) Precision Medicine Working Group has provided recommendations for germline follow‐up testing of variants ascertained during testing of **solid** tumour‐derived deoxyribonucleic acid (DNA).^6^ Their analysis of 49 264 paired tumour‐normal samples indicated that the germline conversion rate of pathogenic variants detected in tumour tissue is high (defined as >10%) for certain genes (such as *BRCA1*, *BRCA2*, *PALB2*), when filtering based on a variant allele fraction (VAF) of 30% (for SNVs) or 20% (for indels) is applied. Comparatively, for certain other genes, such as *TP53*, the germline conversion rate is very low, even after strict filtering based on VAF has been applied, because somatic variation in those genes is a common and early event.

### Solid cancers

When considering variants of putative germline origin picked up through testing of DNA derived from **solid organ** cancers, current UK practice supports follow‐up targeted testing of (likely) pathogenic variants (classified using germline variant interpretation guidelines) in only certain genes, namely, those associated with highest clinical utility and/or with proven association with the cancer type, and only if reported at a VAF of approximately **30%**–**40%** or higher.^4^ There is no equivalent data on haematological cancers.

Irrespective of somatic test results, broader germline testing for cancer may be indicated, depending on the patient's personal and family history, and the eligibility criteria for this are laid out in the NHS National Genomic Test Directory for Rare and Inherited Disease.^5^

### Variant interpretation—context matters

GPV with evidence of high penetrance are considered clinically actionable. People with such variants may be offered irreversible risk‐reducing interventions or pre‐implantation/prenatal testing of at‐risk embryos, while non‐carrier relatives may be reassured and discharged from enhanced surveillance programmes.^7^, ^8^ Participation in any surveillance programme, while not irreversible, also comes with its own risks and benefits. Considering the significant clinical impact on a patient and their family, substantial and consistent evidence for pathogenicity from sources such as population or case–control studies, *in silico* or functional analyses is required for germline variant analysis.^9^, ^10^ In the germline classification system, phenotypic information regarding a patient or from population databases can be used to score for/against pathogenicity. Using phenotype data can be more difficult if population databases contain insufficient detail on phenotype, or there are multiple phenotypes/reduced penetrance associated with a GPV. A highly specific phenotype associated with a particular gene can be used as supporting evidence for pathogenicity. Most haematological cancers, such as acute myeloid leukaemia, which are relatively common in the population, cannot be used to add weight for pathogenicity in this way.

Interpretation of variants picked up in the somatic context aims to determine variant oncogenicity and functional relevance of the variant in ‘driving’ the cancer of interest.^11^, ^12^ Variant oncogenicity, as determined by somatic variant interpretation guidance, is often concordant with, but not equivalent to, variant pathogenicity when constitutional variant interpretation guidelines are applied.^12^, ^13^

### Drivers of myeloproliferative neoplasms (MPN)

The majority of cases of the major myeloproliferative neoplasms (polycythaemia vera [PV], essential thrombocythaemia [ET] and primary myelofibrosis [PMF]) are driven by somatic gain‐of‐function (GOF) variants in one of three genes, *JAK2*, *MPL*, *CALR* ^14^, ^15^ with acquired loss‐of‐function (LOF) variants in *SH2B3* reported in a small minority.^16^, ^17^

While comparatively rare in PV, ET or PMF, GOF variants in *CSF3R* are frequent in chronic neutrophilic leukaemia (CNL) or myelodysplastic/myeloproliferative neoplasm (MDS/MPN) with neutrophilia.^18^

Variants of germline origin in *JAK2*, *MPL*, *SH2B3* and *CSF3R* have been described in the context of myeloproliferative blood indices but the majority tend not, in the absence of other acquired variants, to be associated with a severe disease phenotype, such that the clinical utility of presymptomatic testing in relatives at risk of inheriting such variants is limited. However, in affected individuals, determining whether such a variant is heritable or acquired can help inform the likely clinical trajectory.^19^, ^20^

While monoallelic GOF variants in *MPL* and *CSF3R* are associated with MPN as outlined above, biallelic LOF variants in these genes are associated with autosomal recessive conditions—congenital amegakaryocytic thrombocytopenia (CAMT) and severe congenital neutropenia (SCN) respectively. Identifying a single LOF variant in *MPL* or *CSF3R* during somatic testing of a patient with MPN must be interpreted alongside the phenotype and may represent incidental recessive carrier status rather than a true driver.

### TP53

*TP53* is the single most commonly altered gene in the somatic context and is an unfavourable prognostic indicator in haematological malignancies.^21^ GPV in *TP53* are rare at between 1:3555 and 1:5476^22^ with the exception of one lower penetrance founder variant in the South Brazilian population.^23^ Most GPV in *TP53* confer a high lifetime risk of multiple cancer types (sarcoma, brain tumours, adrenocortical cancer, breast cancer, among others). The ‘classic’ phenotype associated with such variants is described as Li Fraumeni syndrome (LFS), but increasing identification of constitutional *TP53* GPV in patients with atypical or ‘milder’ presentations or *TP53* GPV associated with non‐standard penetrance has given rise to the more inclusive descriptor of ‘heritable *TP53*‐related cancer (hTP53rc) syndromes’.^24^ At present, UK clinical guidelines for management of people with a *TP53* GPV do not distinguish between those who meet the criteria for LFS and those who do not.^25^ Cancer risk, and therefore recommended surveillance, starts from birth. This involves annual whole body and brain magnetic resonance imaging (MRI), abdominal ultrasound between ages 0 and 18 years and breast MRI (for women) from age 20.^25^ The aim of surveillance is early detection of cancer. The psychosocial morbidity associated with the diagnosis and with the intense surveillance programme is significant^26^ although questionnaire‐based evidence from adults in a UK LFS surveillance programme has demonstrated a positive attitude to surveillance and no associated adverse psychological outcomes.^27^

*TP53* is one of the genes commonly implicated in somatic testing for haematological malignancies and is also one of the genes most commonly involved in clonal haematopoiesis (the presence of clonal blood cell populations in otherwise healthy individuals).^28^ It is therefore important to clarify the origin of a variant identified in blood‐derived DNA where a haematological phenotype exists and/or when the phenotype of the patient is inconsistent with constitutional variation in *TP53*.^29^ This may require further testing in a non‐tumour sample, for example, a skin biopsy. Testing multiple sample types and at different time points (e.g. pre‐ and post‐therapy) can be helpful in differentiating between a somatic driver variant, clonal haematopoiesis or a germline variant, including germline mosaicism.

When considering solid organ cancers, germline follow‐up of *TP53* variants of putative germline origin picked up through testing of tumour‐derived DNA is usually only recommended if the patient is younger than 30 years or has a relevant personal or family cancer history. In all cases, germline follow‐up is only recommended when variants are classified as (likely) pathogenic by best practice germline variant interpretation guidance. Interpretation of variants in *TP53* is complicated by the high centrality of the gene, and myriad potential deleterious functional consequences of variation including loss and gain of function, as well as dominant negative effects, and further complicated by the relative rarity of individual constitutional GPV. Application of strict germline variant interpretation guidance for all *TP53* variants identified in the somatic context may be incompatible with desired turnaround times, such that laboratories may not have capacity to implement them in all cases. *TP53* variants may be reported as likely oncogenic in the somatic context, but as variants of uncertain significance when germline ‘rules’ are applied. Discordant classifications pose clinical challenges—particularly when germline follow‐up testing of variants picked up through somatic testing is being considered.

### DDX41

GPV in *DDX41* is the most common genetic predisposition to myeloid neoplasms, found in >3% of adult myelodysplastic syndrome and acute myeloid leukaemia.^30^ Heterozygote status is suspected when somatic NGS testing on a myeloid malignancy finds a *DDX41* variant at a VAF of >40% assessed as LP/P by germline assessment guidelines^9^ sometimes alongside a second (lower VAF, presumed acquired) *DDX41* variant.^31^, ^32^ In this scenario, phenotypic information combined with somatic test results may give rise to high‐level suspicion of germline *DDX41* heterozygosity. A targeted germline test may not be needed for immediate clinical management and NHS workload is a consideration in the numbers of follow‐on targeted germline tests that would be required if all patients in this situation were tested. In the absence of a test confirming germline status however, some laboratories are reluctant to accept samples for predictive testing in relatives, some Haematologists are uncomfortable not referring on to Clinical Genetics, and those patients will not be entered into the new National Inherited Cancer Predisposition Register (NICPR).

A summary of potential benefits and limitations of targeted germline testing of a *DDX41* variant found on somatic testing is shown in Table 1.

**TABLE 1:** Potential benefits and limitations of targeted germline testing of a *DDX41* variant found on somatic NGS testing.

Benefits	Limitations
Patient right to know they have a heritable component to their presentation	High volume, ~3% AML have a germline *DDX41* (likely) pathogenic variant
Unaffected heterozygote relatives can be symptom aware	NHS cost and time implications
Important for transplant donor selection if relatives being matched	Germline test not usually needed for immediate management purposes
Opportunity to collect longitudinal data	Predictive testing for relatives not linked to risk reduction/screening programme
Add to the National Inherited Cancer Predisposition Register	No evidence‐based surveillance guidelines
Opportunity for research and to provide data for possible future evidence‐based management	Impact on Clinical Genetics services and waiting lists if all confirmed heterozygotes referred and cascade testing of relatives initiated

### Copy number variants and other non‐single nucleotide variants

CNVs and other genetic variation that fall outside of SNVs are an emerging issue in terms of how to interpret and manage any germline implications from somatic tests in haemato‐oncology services. Non‐SNVs can include insertions or deletions of >50 base pairs, inversions, tandem duplications, CNVs, rearrangements and translocations.

No single test can identify all types of non‐SNV. NGS read coverage is inherently uneven due to capture biases, which makes raw read counts unreliable for CNV detection. Within the NHS, a combination of techniques is used in haemato‐oncology somatic testing to provide the highest diagnostic accuracy, often including both NGS and cytogenetic array techniques. Detection of non‐SNVs from NGS data is improving via developments in computational analysis.

The aim of these tests is to report non‐SNVs with clinical utility affecting the diagnosis, prognosis and therapy decisions for a patient. Information about copy number variation can also be useful in the interpretation of somatic variants. Analysis of tumour samples may detect constitutional germline CNVs that are common in the general population and not clinically significant, constitutional CNVs that explain or increase risk for the patient's cancer diagnosis or incidental constitutional non‐SNVs that are unrelated to the presenting phenotype. The latter can include changes in the X and Y chromosomes. This may indicate that the patient has a constitutional sex chromosome aneuploidy, for example, Klinefelter syndrome (47,XXY) or XYY syndrome in men and Turner syndrome (e.g. 45,X) or Triple X syndrome (47,XXX) in women.

Germline non‐SNVs cannot be definitively called from data derived from somatic testing in diseased blood or bone marrow. Filtering is key to avoid generating unnecessary concern regarding the possibility of germline origin of non‐SNVs. Laboratory reports cannot provide clear germline results from these somatic tests but may include information about non‐SNVs with a comment that germline origin cannot be excluded. Ideally, this process would include phenotypic information and family history to aid reporting, although this practice is currently variable. There is a need for non‐SNV detection and reporting to be standardised across NHS services.

### National inherited cancer predisposition register

On 1 July 2025, the National Disease Registration Service (NDRS) launched the National Inherited Cancer Predisposition Register (NICPR) for England.^3^ This registry collects data on individuals with genetic predispositions to cancer in England. It expands on previous systems like the National Lynch Registry ^33^ to include more cancer susceptibility genes. The register currently includes ~120 cancer predisposition genes for which testing is currently available within the NHS National Genomic Test Directory for Rare and Inherited Disease.^5^ The NICPR will be able to incorporate additional genes if testing becomes available in the future. The remit of the NICPR is to develop a dynamic real‐time register of all individuals with a known germline predisposition to cancer. Plans are underway to link NICPR patient data to national screening programmes, for example, the Very High Risk breast surveillance programme for *BRCA1/BRCA2/PALB2* heterozygotes.^34^ The NDRS can link genomic data with other clinical data, such as cancer diagnoses, over significant timeframes (decades). This facilitates rich linked longitudinal genotype–phenotype datasets often extremely difficult to capture through research due to the time‐limited nature of grant funding. NHS Clinical Genetics services are responsible for adding patients to the register. Over time, the NICPR will create further opportunities for entry to cancer surveillance programmes, clinical audit, service evaluation, evidence‐based penetrance estimates and research.

## METHODS

### Pre‐meeting preparation

The organising committee comprised seven NHS health professionals based in different regions of England representing Haematology, Clinical Genetics and Clinical Scientists, from three national collaborative groups: the UK Cancer Genetics Group (UKCGG), the Cancer Research UK (CRUK) funded CanGene‐CanVar research programme (CGCV) and the NHSE Haematological Oncology Working Group.

The specific topics were chosen as the focus of this meeting based on the organising committee's experience in clinical practice, the existence of research evidence that could form the basis of potential consensus decisions and new developments affecting patient pathways. A literature review informed the background reading document which was prepared by the organising committee and circulated to all registered delegates in advance of the meeting.

Invitations were sent to each Genomics Laboratory Hub (GLH) within NHS England, plus the Regional Genetics Services in Scotland, Northern Ireland and Wales, alongside haematological services in each GLH region and policy‐makers from NHS England. Representatives from the following NHS specialities were encouraged to register: Haematology, Clinical Genetics and Clinical Scientists.

Three charities supporting patients with blood cancer were approached and agreed to participate. These were Leukaemia Care, MPN Voice and MDS‐UK.

Prior to the meeting, a scoping survey (Appendix S1) was sent to registered delegates. This approach was successful in the first national consensus meeting on genetic testing in haematological malignancies.^1^ The survey questions aimed to assess current practice and seek opinion on potential best practice pathways. The themes arising from the survey were used to create a series of key questions to be addressed at the meeting.

### Consensus group participants

A total of 217 stakeholders registered to attend from across the United Kingdom, comprising Clinical Geneticists, Genetic Counsellors, paediatric and adult Haematologists, Haematopathologists, Clinical Scientists (somatic and germline) and patient representatives. Each of the seven GLHs within NHSE were represented as well as delegates from Scotland, Wales and Northern Ireland. Colleagues from Hong Kong and Australia were also present as observers and to give an international perspective where appropriate but did not participate in the polling. Consensus meeting attendees are listed in Appendix S2.

### Workshop format

The meeting agenda is available in Appendix S3. Each topic was introduced by an invited expert speaker in a structured series of talks. These covered the survey results, an educational background session, laboratory pathways for somatic and germline testing, *DDX41*, *TP53*, CNVs/non‐SNVs and MPN.

These presentations provided a review of the available evidence and equipped the delegates from a variety of backgrounds with up‐to‐date evidence on which to base their recommendations. Slides and recordings from the day are available at: https://www.ukcgg.org/information‐education/ukcgg‐consensus‐meetings/.^35^

Thereafter, a number of related polls were conducted, with proposed statements for best practice in different scenarios. Each poll was closed when at least half of delegates present had submitted a response. In practice, this number changed based on the number of participants in the meeting, as not all registered participants could attend in full on both days (participants day 1 = 159, day 2 = 139). It was anticipated that delegates would sometimes choose to abstain from voting on specific scientific or clinical questions that were outside of their area of expertise. Consensus was deemed to be reached when ≥80% respondents selected ‘Agree/Strongly Agree’ in response to a statement. Within the Delphi process, it is important to set a consensus level at the beginning of the process.^1^ If an argument was proposed requiring revision to the wording of the statement, this was undertaken in ‘real time’ and the poll was repeated with revised wording to generate a final decision. Time was allocated for whole group discussion around each polling question, which helped inform any consensus reached. After the meeting, a summary was posted on the UKCGG website.

A follow‐up workshop was organised to focus on *DDX41* on 7 October 2025, as limited consensus was reached on this topic in the April 2025 meeting. The outcomes of the discussions and polling over both meetings (April and October 2025) are included in this article.

## RESULTS

The pre‐meeting survey was sent to all registered delegates and open for submissions between 20 January 2025 and 14 February 2025. At the time the survey was closed in February to allow time for data analysis to present at the consensus meeting, there were 49 responses from 164 registered delegates (30%). Respondents included Haematologists (adult and paediatric, *n* = 14), Clinical Geneticists/Genetic Counsellors (*n* = 15), Clinical Scientists (somatic or germline, *n* = 18), a patient/charity representative (n = 1) and a researcher (n = 1).

Responses to the pre‐meeting survey were received from six of the seven GLHs in England as well as Scotland and Northern Ireland. Of 49 respondents, 28 (57%) stated that they participated in the first national consensus meeting on germline predisposition to haematological malignancies in 2022. Results from the pre‐meeting survey showed that the majority of targeted germline testing as follow‐up to diagnostic somatic panel results is organised within haematology, and that challenges remain regarding the interpretation of potential germline findings, with respect to VAF thresholds, variants of uncertain significance, incidental findings and heterozygous findings in recessive genes. Survey results were used to inform the consensus meeting agenda and to draft consensus statements for discussion and polling.

Statements on which consensus was reached as best practice guidelines are shown in Table 2. Further comments on each section follow afterwards using the same section headers.

**TABLE 2:** The statements on which consensus was reached as best practice guidelines.

Genomics MDT working and variant reporting It would be best practice for clinicians & scientists to have access to an MDT with somatic and germline Clinical Scientists, Haematologists and Clinical Genetics to discuss genomic results which may be heritable in haemato‐oncology patients. **(Agree/Strongly agree: 99%)** In order to perform full evidence‐based interpretation of a variant of potential germline origin identified on DNA extracted from peripheral blood or bone marrow, additional clinical/phenotypic data may be required at time of reporting. **(Agree/Strongly agree: 94%)** If relevant clinical data are unavailable at time of reporting due to time constraints, it would be best practice to report that further clinical information/MDT discussion would be required for decisions around germline classification and confirmation. **(Agree/Strongly agree: 92%)** It would be best practice to have an MDT discussion regarding a genomic variant before germline confirmation if the variant has an unlikely or no known association with the presenting phenotype. **(Agree/Strongly agree: 87%)** A statement on the report suggesting possible germline origin of a variant should be considered for any variant where a confirmed germline finding would have potential clinical significance (especially if the VAF is >40%). **(Agree/Strongly agree: 81%)** A statement on the report suggesting possible germline origin of a variant is not routinely required where VAF <35% unless there is other supporting evidence for potential germline origin. **(Agree/Strongly agree: 81%)**
*DDX41* 7 A *DDX41* variant in tumour DNA (germline ACMG class LP/P) with VAF ≥35% with a second possible driver (lower VAF, presumed acquired) variant, can be presumed to be likely germline origin for immediate diagnostic/management purposes. **(Agree/Strongly agree: 90%)** **When it is suspected that an identified variant in *DDX41* (LP/P in germline context) is germline in origin (scene setting on polling for statements 8–12):** 8 Where resources allow, it is reasonable to offer a germline test in this situation. **(Agree/Strongly agree: 95%)** 9 Where a target germline test has not been undertaken, it is best practice to document in the report that the *DDX41* variant could be present in the germline. **(Agree/Strongly agree: 99%)** 10 When confirmatory germline testing is not possible, predictive genetic testing can be offered to at‐risk family members based on a somatic report, after counselling regarding the lack of evidence‐based surveillance for carriers of *DDX41* variants. **(Agree/Strongly agree: 85%)** 11 It is best practice to refer patients with a confirmed *DDX41* germline variant (ACMG class LP/P) to Clinical Genetics for genetic counselling and discussion of implications for the patient and wider family. **(Agree/Strongly agree: 97%)** 12 If surveillance of some type is to be offered, this should be in the context of a research study/programme, to facilitate collection of longitudinal data on a prospective basis. **(Agree/Strongly agree: 94%)**
*TP53* 13 Given the low somatic to germline conversion rate for *TP53*, VAF should not be considered in isolation when deciding if it is necessary to offer germline testing to patients who have a somatic LP/P *TP53* variant. **(Agree/Strongly agree: 93%)** 14 It would be best practice to consider patient age, personal and family history, plus clinical/molecular/pathological features of the haematological malignancy and treatment pathways when deciding if targeted germline *TP53* testing is required. **(Agree/Strongly agree: 100%)** 15 It would be best practice to discuss patients in whom targeted germline *TP53* testing is being considered with a multidisciplinary team including Clinical Genetics prior to undertaking germline testing of a tumour‐detected *TP53* variant. **(Agree/Strongly agree: 93%)** 16 It would be best practice to inform patients undergoing germline testing of a tumour‐detected LP/P *TP53* variant of the clinical consequences of identifying this in germline for them and their family in advance of the germline test. **(Agree/Strongly agree: 92%)** 17 If urgent testing for a potential germline LP/P *TP53* variant is being offered to relatives for the purposes of bone marrow transplant testing pathways, it is best practice to offer the relative access to pretest genetic counselling. **(Agree/Strongly agree: 99%)** 18 Targeted germline testing should not be undertaken for *TP53* variants classified by germline ACMG guidelines as a Class 3 VUS unless there is a strong clinical rationale for testing and only with MDT agreement including Clinical Genetics. **(Agree/Strongly agree: 100%)** 19 If testing for a potential germline *TP53* VUS in a proband, it is best practice to offer pretest genetic counselling with an expert in hereditary cancer such as a Genetic Counsellor, Clinical Geneticist or other trained specialist practitioner. **(Agree/Strongly agree: 95%)** 20 If testing for a potential germline *TP53* VUS is being offered to relatives due to bone marrow transplant pathways, it is best practice to offer the relative pretest genetic counselling with an expert in hereditary cancer. **(Agree/Strongly agree: 98%)**
Non‐SNV variation 21 It is best practice where a CNV of possible germline origin is identified to confirm whether the CNV would be reportable in the germline setting before highlighting this on the report as of possible germline origin. **(Agree/Strongly agree: 93%)** 22 Scientists should ideally have pre‐reporting access (via MDTs or other routes of communication) to germline scientific/clinical expertise when deciding if CNVs of uncertain significance of potential germline origin should be reported. **(Agree/Strongly agree: 98%)** 23 If a CNV of potential germline origin not causative of the phenotype but relevant for other disease risks is identified, it would be best practice to discuss with Clinical Genetics prior to referring patients for germline testing. **(Agree/Strongly agree: 96%)** 24 If a CNV of potential germline origin not causative of the phenotype but relevant for other disease risks is identified, BSGM guidance on incidental findings should be followed when reporting CNVs containing cancer susceptibility genes. **(Agree/Strongly agree: 94%)** 25 Consider obtaining further phenotypic information and/or MDT discussion before reporting aneuploidy/sex chromosome abnormalities with significant clinical consequences with suspicion of germline origin. **(Agree/Strongly agree: 97%)** 26 Aneuploidy/sex chromosome abnormalities which may have significant clinical consequences (e.g. X/O or XXY) should go to an MDT discussion to decide appropriate follow‐up. **(Agree/Strongly agree: 92%)** 27 Sex chromosome abnormalities/aneuploidy of possible germline origin identified on tumour testing which would have limited clinical consequences (e.g. XXX or XYY) will not necessarily require reporting/further follow‐up. **(Agree/Strongly agree: 85%)** 28 When aneuploidy/sex chromosome abnormalities with suspicion of germline origin with significant clinical consequences have been reported consider obtaining further phenotypic information and/or MDT discussion to ensure appropriate clinical follow‐up. **(Agree/Strongly agree: 98%)**
Myeloproliferative neoplasms 29 Where required, *JAK2*/*MPL*/*SH2B3* targeted germline testing for a myeloproliferative phenotype can be performed within haematology and does not require a referral to Clinical Genetics. **(Agree/Strongly agree: 92%)** 30 Clinical Genetics should not routinely accept referrals for cascade testing of *JAK2*/*MPL*/*SH2B3* germline variants for a myeloproliferative phenotype unless there is a clear clinical rationale or relevant national guideline. **(Agree/Strongly agree: 98%)** 31 Germline testing of heterozygous variants in the context of a mild myeloproliferative phenotype, where results will not inform clinical management, is not routinely required. **(Agree/Strongly agree: 95%)** 32 If after GTAB/MDT consideration it is felt that an inactivating *MPL*/*CSF3R* variant is consistent with carrier status for an AR condition (i.e. unlikely causal for patient phenotype), this does not need to be reported. **(Agree/Strongly agree: 87%)** 33 It would be good practice for laboratories to maintain a central list of carriers of probable germline *JAK2*, *MPL* and other MPN‐related gene variants which may be contributing to a myeloproliferative phenotype for national registries when available. **(Agree/Strongly agree: 98%)** 34 Non‐canonical variants in *JAK2* with an allele frequency suggestive of them being heterozygous germline variants which are suspicious for being causal of the phenotype should be reported. **(Agree/Strongly agree: 86%)** 35 It is reasonable to test germline status for a non‐canonical *JAK2* variant with an allele frequency suggestive of being a heterozygous germline variant, where there is potential evidence of causality, in order to inform the likely clinical trajectory. **(Agree/Strongly agree: 81%)** 36 The primary clinical reason for offering *JAK2* targeted germline testing is to define the clinical entity and disease course rather than to determine familial risk and offer downstream cascade testing. **(Agree/Strongly agree: 82%)** 37 It is best practice to inform patients with a confirmed germline LP/P *JAK2* variant that the variant may be present in their relatives and could cause a similar phenotype. **(Agree/Strongly agree: 92%)** 38 Patients should be advised that first‐degree adult relatives of a person with a confirmed germline LP/P *JAK2* variant can be offered an FBC. A relevant FBC abnormality should prompt a referral to a Haematologist. **(Agree/Strongly agree: 90%)** 39 Premature terminating variants in the *MPL* gene should be interpreted in conjunction with the phenotype (i.e. proliferative or cytopenic). **(Agree/Strongly agree: 83%)** 40 Variants in the *CSF3R* gene should be interpreted in conjunction with the phenotype (i.e. proliferative or cytopenic). **(Agree/Strongly agree: 98%)** 41 It is reasonable to report single nucleotide variants in *SH2B3* with an allele frequency suggestive of being a heterozygous germline variant, where there is potential contribution to phenotype, in order to define the clinical entity and disease course. **(Agree/Strongly agree: 96%)** 42 It is reasonable to perform targeted germline testing for a likely heterozygous *SH2B3* variant, which is thought to be contributing to a clinically significant phenotype, in order to define the clinical entity and disease course. **(Agree/Strongly agree: 94%)**
General statement 43 Further national research and clinical initiatives are required to progress this work **(Agree/Strongly agree: 98%)**

Abbreviations: ACMG, American College of Medical Genetics and Genomics; AR, autosomal recessive; BSGM, British Society of genetic Medicine; CNV, copy number variation; FBC, full blood count; GTAB, Genomics Tumour Advisory Board; LP/P, likely pathogenic or pathogenic; MDT, multidisciplinary team or an MDT meeting; VAF, variant allele fraction; VUS, variant of uncertain significance.

## DISCUSSION

### Genomics MDT working and variant reporting

It was clear from the pre‐meeting survey and discussions that most delegates have access to an MDT or GTAB and use that forum to discuss potential germline implications of NGS testing in haemato‐oncology patients. MDT working involves contribution from the different staff groups involved, ideally including clinicians who can provide phenotypic information. In practice, provision of phenotypic information on test forms may lack detail and the time constraints of busy NHS clinicians to attend MDT meetings means phenotypic information may be limited. In addition, the laboratories are required to meet rapid turnaround times for somatic reports needed for diagnostic purposes, which can hamper the ability to fully interpret a potential germline finding. Comments from Clinical Scientists in the pre‐meeting survey acknowledged this time pressure as well as the value of clinician input and phenotypic information within and outside of an MDT meeting.

In line with the discussions from the first national consensus meeting in 2022,^1^ delegates did not feel it was possible or helpful to have a strict VAF threshold for targeted germline testing. The most common response in the pre‐meeting survey question suggested a majority of laboratories include a statement that a variant may be of germline origin for pretreated diseased blood/bone marrow on somatic (tumour only) testing when the VAF is 30% or more, as per ESMO guidance for solid tumours.^6^ In the pre‐meeting survey, the most common response to the question about preferred VAF threshold to use before considering targeted germline testing was 40%. There was a lively discussion about this during the consensus meeting, with an agreement that VAF thresholds should not be used in isolation for this decision.

### Gene‐specific considerations

#### DDX41

In the meeting on 3 and 4 April 2025, consensus was reached only on a single statement about *DDX41* (statement number 7). There was debate on the clinical utility of targeted germline testing and cascade testing, outside of transplant considerations. Some delegates expressed concern about communication of results and implications of a presumed but unconfirmed germline *DDX41* variant. At a subsequent *DDX41*‐focussed meeting on 7 October 2025, the following question was addressed: ‘Under what circumstances should targeted germline testing be undertaken for somatic *DDX41* variants?’. Additional consensus was reached on the situation where suspicion is high that an identified variant in *DDX41* (LP/P in germline context) on a somatic panel is germline in origin (statements number 8–12).

#### TP53

High‐level consensus was reached on the proposed statements on *TP53* with limited revision to the wording required. Given the clinical and psychosocial impact of a diagnosis of Li Fraumeni syndrome in a family, it was agreed that there should be careful consideration before targeted germline testing, with multiple factors feeding into this decision. Phenotypic information is key, with information on previous cancer history and a patient's family history informing the discussion at MDT. Discussion in the meeting focussed on the situation in which there are discordant classifications in the somatic and germline settings, and the management of a potential germline VUS. Targeted germline testing and cascade testing in relatives would not usually be carried out for a VUS. Special consideration in haemato‐oncology clinics is needed when a relative is being considered as a potential allogeneic bone marrow transplant donor. This is because relatives with germline variants predisposing to haematological malignancies should not be used as donors where suitable alternatives are available.^2^ In this complex and time‐sensitive scenario, it was agreed that best practice for MDT working involves Clinical Genetics, with pretest genetic counselling provision for relatives.

### Non‐SNV variation

The pre‐meeting survey showed variability in the degree to which CNVs and non‐SNVs are an issue in somatic reporting of haemato‐oncology tests. The expert speaker conveyed the difficulty in precise determination of CNVs as well as the importance of detecting constitutional CNVs that explain or increase risk for the patient's presenting phenotype. This informed the meeting discussion and polling on other potentially germline non‐SNVs which may be suggested by the somatic data. Many of these, even if confirmed as germline may not be reportable (i.e. clinically significant) from a germline cytogenetics perspective. The polling results supported somatic Clinical Scientists having access to colleagues with expertise in germline non‐SNVs, from the laboratory and Clinical Genetics services. High‐level consensus was reached that not all potentially germline sex chromosome aneuploidies need to be reported and that these decisions should focus on clinical actionability.

### Myeloproliferative neoplasm susceptibility genes

The majority of MPNs are considered sporadic although research evidence has suggested shared genetic susceptibility.^36^ The diagnostic utility of germline testing for variants found in *JAK2*, *MPL*, *CALR*, *SH2B3* and *CSF3R* somatic testing may be primarily to exclude somatic status, rather than to confirm germline status. Where there is research evidence of a phenotype associated with an LP/P variant in one of these genes, high‐level consensus was achieved on advice about a full blood count in relatives, with input from Haematology if abnormal (*JAK2*, statements 34–38, *SH2B3* statements 41–42). These statements are not exhaustive and other testing scenarios may also be appropriate (e.g. *JAK2* statements may also be relevant to *MPL* variants thought to be clinically significant). In practice, this information could be provided by a Haematologist to their patient to pass on to close adult relatives, with abnormal full blood count in primary care prompting a referral for the relative into Haematology.

Further research is needed on the association of germline variation in MPN‐associated genes and non‐malignant haematological phenotypes. At present within the NHS, cascade testing of *JAK2*, *MPL*, *CSF3R* and *SH2B3* germline variants is not routinely performed after targeted testing confirms germline status after diagnostic somatic NGS.

For *MPL* and *CSF3R*, identifying biallelic LOF variants may reveal a significant diagnosis of an autosomal recessive condition, while identification of a monoallelic LOF variant is likely consistent with carrier status rather than a contributory driver for MPN, considering that GOF variants in such genes are more relevant for this phenotype.

Phenotypic information is crucial in assessing the significance of finding one LOF variant of possible germline origin in these genes—as is robust assessment of data demonstrating loss or gain of function. Likely germline heterozygous variants that do not correlate with the patient's phenotype can be considered incidental findings. In UK clinical practice, there are clinical practice guidelines for incidental findings.^37^ These acknowledge the increasing likelihood of finding autosomal recessive carrier status with the expansion of genetic testing in the NHS. In these guidelines, reporting of autosomal recessive carrier status is not recommended as a default approach when unrelated to the phenotype. In clinical practice, phenotypic information may not always be specific and well defined, which means consideration is given to the test sensitivity and the possibility of a ‘missed’ second variant.

Within NHS England, decisions around reporting incidental carrier status are also shaped by the Genomic Test Directory.^5^ The ‘R246 Carrier testing at population risk for partners of known carriers of nationally agreed autosomal recessive disorders’ panel stipulates that the decision on whether to report a finding of incidental carrier status depends upon whether cascade testing would be offered for relatives and their partners. This should take into consideration the disease prevalence (>1 in 70 carrier frequency in the relevant population), whether there are common variants that could be tested and whether there is known consanguinity. Given the context in which somatic testing occurs for suspected haematological malignancy, it was recognised that consanguinity may not be known at the time of reporting.

Further work planned or underway relating to haematology–oncology NHS pathways is described in Table 3.

**TABLE 3:** Further work planned/underway related to haematology–oncology NHS pathways.

Further work	Comments
To request the addition of *SH2B3* to relevant panels on the Rare and Inherited Disease Genomic Test Directory	Application submitted by UK Cancer Genetics Group in June 2025 for addition to R406 Thrombocythaemia panel in the National Genomic Test Directory
To build the **UK Ra**re **M**yelo**P**roliferative **Var**iant Registry	Ongoing
To improve capture of haemato‐oncology patients and their relatives who have had genetic testing on the National Inherited Cancer Predisposition Register (NICPR)	Ongoing
To carry out a national audit on NHS Genomic Laboratory Hub data to assess germline conversion rates at different VAFs for SNVs and CNVs	Not started yet
Submit expression of interest for a new workstream in the existing Haemato‐oncology NHS Genomics Networks of Excellence	Expression of interest submitted 26/11/2025. Business case submitted 22/12/2025 for new workstream ‘National Haemato‐Oncology Predisposition and Variant Interpretation Network of Excellence (HOP‐VINE)’

### Areas where consensus was not reached

There were two statements which had high‐level agreement but did not meet the consensus threshold:

In the absence of evidence‐based surveillance for unaffected *DDX41* heterozygotes, there is no expectation for routine follow‐up **(Agree/Strongly agree: 77%)**.

Further work is required to develop the evidence base to inform future management approaches.

If somatic testing identifies a *DDX41* VUS of likely germline origin without a co‐occurring somatic variant, it is not necessary to routinely report if the phenotype is unlikely to be *DDX41*‐associated (after discussion at MDT) **(Agree/Strongly agree: 76%)**.

### Summary

This was the second UK meeting to address clinical and laboratory pathways for patients with suspected or confirmed haematological malignancy. The strength of the guidance derives from UK‐wide invitation (no number cap for the virtual meeting or disadvantage due to travel). The key issues were selected for the agenda based on seeking opinion of stakeholders prior to the meeting, and there was attendance from broad specialities including patient advocates. The different perspectives and expertise of the group enriched the discussion and enabled the group to achieve consensus views. This meeting further facilitated the collaborative, multidisciplinary efforts in the United Kingdom to standardise and improve clinical and laboratory pathways in haemato‐oncology NHS care.

## AUTHOR CONTRIBUTIONS

All named authors were part of the organising committee and/or contributed significantly to the planning, delivery or manuscript preparation. All named authors have reviewed the manuscript and approved this for submission. Katie Snape and Terri P. McVeigh—conceptualisation, organisation/delivery of meeting and review of manuscript. Beverley Speight—organisation/delivery of meeting, oversight of process, writing first draft and editing with co‐author comments. Angela Hamblin and Polly Talley—organisation/delivery of meeting and review of article. Jamshid Khorashad, Austin Gladston Kulasekararaj, Rachel Robinson, Anna Godfrey, Adam Mead and Phil Dean—contribution to meeting and review of the article.

## FUNDING INFORMATION

KS supported by Cancer Research CRUK Catalyst Award, CanGene‐CanVar (C61296/A27223). ALG—Advisory board, speaker honoraria and conference support from Novartis; consulting for Johnson & Johnson; speaker honoraria from MD‐Education.

## CONFLICT OF INTEREST STATEMENT

No conflicting/competing interests declared.

## Supporting information


Data S1.

## Data Availability

Data sharing not applicable to this article as no datasets were generated or analysed during the current study.
