# Supplementary material for: Somatic and germline genetic testing pathways in haematological malignancies: Best practice consensus guidelines from the 2025 national meeting organised by UK Cancer Genetics Group (UKCGG), CanGene‐CanVar and the NHS England Haematological Oncology Working Group
Source: Br J Haematol. 2026 Mar 3;208(4):1211–22. doi: 10.1111/bjh.70404 (PMC13071493; doi:10.1111/bjh.70404)
Supplement: Supplementary file 1 — Data S1. [file BJH-208-1211-s001.docx]

**Supplementary material**

**Appendix 1: Pre-meeting survey questions**

What is your role?

If applicable, which Genomics Laboratory Hub/region do you work in?

Did you attend the first national consensus meeting on genetic testing in haematological malignancies in April 2022?

Do you have access to an MDT to discuss somatic and germline results for haemato-oncology patients?

Who attends this meeting on a regular basis?

How often do you review a case at your MDT where there is insufficient phenotypic information to support the discussion?

How often do you review a case at your MDT in which there are findings discussed that are unlikely or not known to be relevant to the presenting phenotype?

At present in your region, what Variant Allele Fraction(VAF) is currently used by the laboratory to include a statement that the variant may be of germline origin for pre-treated (at diagnosis) diseased blood/bone marrow on somatic (tumour-only) testing?

In the majority of routine cases, what would be your preferred minimum threshold for triggering the consideration of a potential germline origin?

At present in your region, do you routinely include information about a Variant of Uncertain Significance (VUS) that may be of germline origin in a test report?

If you think a variant is of germline origin, in which of the following situations would you report a VUS? (routine cases) – matrix question on VUS with very limited evidence towards actionability in a gene related to phenotype / gene not known to be related to presenting phenotype.

If you think a variant is of germline origin, in which of the following situations would you report a VUS? (urgent cases related to bone marrow transplant where relatives are potential donors) - matrix question on VUS with evidence pointing towards actionability in a gene related to phenotype / gene not known to be related to presenting phenotype.

If you think a variant is somatic (acquired, not in germline), in which of the following situations would you report a VUS? (routine cases) ) – matrix question on VUS with very limited evidence towards actionability in a gene related to phenotype / gene not known to be related to presenting phenotype.

If you think a variant is somatic (acquired, not in germline) in which of the following situations would you report a VUS? (urgent cases related to bone marrow transplant where relatives are potential donors) - matrix question on VUS with evidence pointing towards actionability in a gene related to phenotype / gene not known to be related to presenting phenotype.

In your region, for the majority of routine cases what sample type would be used most often to determine germline status for DDX41, GATA2, RUNX1, CEBPA, ANKRD26, ETV6

Please consider the following scenario. In DNA from bone marrow in an affected patient you identify two DDX41variants, one at low VAF consistent with being acquired and another likely pathogenic/pathogenic (Class 4/5) variant at a VAF of over 40%. A remission sample is sent from this patient in which the likely pathogenic/pathogenic (Class 4/5) variant remains present at a VAF consistent with germline origin. Would you consider this acceptable to report as very likely to be a germline variant?

Please consider the following scenario. In DNA from bone marrow in an affected patient you identify a single likely pathogenic/pathogenic (Class 4/5) variant at a VAF over40% in a gene in which there is a recessive germline phenotype (i.e. biallelic variants are required to cause the phenotype). Would you discuss doing further investigations in this patient at MDT?

Do you apply different criteria when assessing whetherTP53 somatic variants may be of germline origin compared to other genes (e.g. RUNX1) in haemato-oncology patients in your region?

When a TP53 variant is detected on a somatic panel, what factors do you routinely consider to assess if germline testing is indicated? (select all that apply in your current practice – ignore it / discuss at MDT / report it / refer to Clinical Genetics / other)

In your region who does the majority of pre-test counselling/consenting when germline testing is arranged for a variant found on somatic testing?

Are you finding loss of heterozygosity (LOH) on arrays or WGS tests done in the diagnostic haemato-oncology test pathways that lead to discussions about whether it could be a germline copy number variant?

If you identify a copy number variant of potential germline origin after somatic testing, what do you do in current practice?

Please state the three issues that are causing the most challenges in your Haemato-oncology pathways relating to potential germline findings from somatic testing.

**Appendix 2 List of meeting attendees**

Attendee list for 3^rd^ / 4^th^ April

Encarna Gomez-Garcia, Mireia Atance, Bianca DeSouza, Bridget Manasse, Katie Snape, Hood Mugalaasi, Dan Lock, Amy Logan, SARA PERLADO, Terri McVeigh, Beverley Speight, Angela Hamblin, Caoimhe Egan, David Clarke, Pooja Dasani, Joana Gomes, Andrew McGregor, Ana Oliveira, Rachel Robinson, Nahid Kamal, Polly Talley, Laura Chiecchio, Austin Kulasekararaj, Anjum Khan, Sarah Westbury, Charlotte Crowley, Sian Lewis, Teresa Arquero, Omaima Ahmed, Manoj Raghavan, Richard Chasty, Lucy Side, Claire Searle, Anna Godfrey, Helen Hanson, Chris Wragg, Paula Waits, Kate Musgrave, Rebecca Collier, Olga Tsoulaki, Laura Elmhirst, Alice Wong, Kate Moloney, Kevin Baker, Jessica Charlton, Chloe Cassidy, Vicky Gkreka, Clare Bryant, Nancy Whish, Carryl Dryden, Gonzalo Castellanos, Jamshid Khorashad, Yasmin Reyal, Monisha Shanmugasundaram, Kar Lok Kong, Zoe Thorn, Tara Clancy, Oluwatosin Taiwo, Andrew Innes, Ringo Lam, Kelly Kohut, Alistair Reid, Dushy Navarajasegaran, Victoria Campbell, Jessica Gabriel, Hannah Musgrave, Phil Ostrowski, Philip Dean, Luke Carter-Brzezinski, Jon Mathias, Debbie Hickman, joanne mason, Charlotte Attwood, Jitha Vijayakumar, nicholas Lea, Ilenia Simeoni, Rachel Mayhew, Julian Cano-Flanagan, Ana Rio-Machin, Adele Timbs, Guy Hannah, Julie Young, Angela Brady, Clare Crean, Andrew Green, Helen Warren, Elizabeth Ratsma, Robert Dunn, Paul Warman, Claire McKeeve, Sally Spillane, Jack Corfield, Anthony Bench, Katie Jones, Ellen Higgs, Azim Mohamedali, Deirdre Donnelly, Nick Parkin, Kai Ren Ong, Dorte Wren, Catherine Cargo, Iryna Stasevich, Dorothy Halliday, Jackie Cook, Steve Best, Nina Orfali, Marc Tischkowitz, Rosalyn Jewell, Matt Cullen, Timothy Chevassut, Josephine Goodwin, Alison Callaway, Sahla Ali, Marie-Claire Smith, Yiwen Liu, Lily Barnett, Rachel Moore, Laura Kingham, Jennie Murray, Luke Ficinski, Joanna Large, Yasmin Clinch, Kath Smith, Emily Mitchell, Aytug Kizilors, Maartje Nielsen, Kiran Tawana, Anupama Rao, Phil Ancliff, Sangeeta Awal, Clair Engelbrecht, Iman Al Noumani, George Vassiliou, Thais Ferrari Gersogamo, Alameldin Abdallah, Francesco Versino, Sandi Deans, Claire Schwab, Mark Catherwood, Rachel Wells, Ingrid Simonicova, Audrey Morris, Fran Aldridge, Sita Ram, Sara Ribeiro, Joseph Christopher, Shreyans Gandhi, Andrew Hindley, Malee Fernando, Encarna Gomez, Livia Raso-Barnett, Jack Bartram, Maia Hickin, Megan Mitchell, Paul James

Attendee list for 7^th^ Oct

Beverley Speight, Rachel Robinson, Olga Tsoulaki, Polly Talley, Terri Mcveigh, Angela Hamblin, Ilenia Simeoni, Kim Reay, Katherine Smith, Nancy Whish, Rosemarie Davidson, Helen Hanson, Phil Ostrowski, Caroline Shak, Hannah Musgrave, Steven Best, Rebecca Collier, Jamshid Khorashad, Julia Baptista, Claire Mckeeve, Alison Callaway, Kate Moloney, Laura Elmhirst, Clare Crean, Jennie Murray, David Clarke, Anthony Bench, Amy Rachel Moore, Maria Lara Fanego, Juliee Mcgimpsey, Rosalyn Jewell, Andrew Hindley, Nicola Austin, Elizabeth Ratsma, Malee Fernando, Nicholas Lea, Jack Corfield, Jackie Cook, Monika Domeradzka, Kar Lok Kong, Livia Raso-Barnett, Maia Hickin, Clair Engelbrecht, Nahid Kamal, Bianca Desouza, Jonathan Campbell, Jessica Charlton, Aytug Kizilors, Abhishek Dashora, Andrew George, Manoj Raghavan, Shreyans Gandhi, Molly Thompson, Austin Kulasekararaj, Alesia Khan, Kevin Baker, Mike Dennis, Audrey Morris, Joana Gomes, Amber Bryce, Guy Hannah, E. Gomez, Alistair Reid, Liron Barnea Slonim, Timothy Chevassut, Aditi Vedi, Frances Smith, Steven Hardy, Deirdre Donnelly, Varisha Desai, Arief Gunawan, Claire Searle, Daniel Lock, Rebecca Pollitt, Catherine Cargo, Caoimhe Egan, Oluwatosin Taiwo, Anjum Khan, Roochi Trikha, Mireia Atance Pasarisas, Victoria Campbell, Sara Perlado Marina, Eleanor Carden, Isabel Sa, Dushanty Navarajasegaran, Sarah Westbury, Rachel Mayhew, Manish Jain, Monisha Shanmugasundaram, Rhianna Rakhra, Bridget Manasse, Smitha Nevis, Andrew Innes, Amy Logan, Helen Warren, Ingrid Simonicova, Frances Aldridge, Azim Mohamedali, Kiran Tawana, Eirini Oikonomidou, Elisabeth Rolf, Rachel Wells, Kai Ren Ong, Paula Waits

**Appendix 3: Agenda from 3^rd^/4^th^ April 2025 meeting**

**Somatic to germline genetic testing in haematological malignancies**

**2nd National Consensus Meeting**

**Agenda**

**Thursday 03 April, 9:15am to 12:45pm**

| 09:15 to 09:35 | **Introduction to meeting: welcome, housekeeping, pre-meeting survey results**  **Bev Speight** |
| --- | --- |
| 09:35 to 09:55 | **Educational session:** variant types in the somatic/germline setting, inheritance patterns, SNVs/CNVs, Haem-Onc capture in the National Inherited Cancer Predisposition Register  **Dr Katie Snape** |
| 09:55 to 10:15 | **Recap of pathways from previous consensus meeting, update on post-implementation issues, focus on DDX41**  **Dr Terri McVeigh** |
| 10:15 to 10:30 | **Incorporation of germline confirmatory testing into Haem-Onc diagnostic pathways**  **Dr Rachel Robinson** |
| 10:30 to 10:45 | **How one GLH has managed the issues and pathways so far**  **Dr Phil Dean** |
| 10:45 to 11:20 | **Consensus statements and polling**  **Chaired by Dr Polly Talley** |
| 11:20 to 11:45 Break | |
| 11:45 to 12:10 | **TP53: detection in Haem-Onc setting, penetrance, surveillance, reference to restrictive ESMO guidelines, with case study**  **Dr Terri McVeigh** |
| 12:10 to 12:45 | **Consensus statements and polling**  **Chaired by Dr Terri McVeigh** |

**Friday 04 April, 09:15am to 12:45pm**

| 09:15 to 09:25 | **Welcome to meeting**  **Bev Speight** |
| --- | --- |
| 09:25 to 09:55 | **Non-SNV variation in Haem-Onc somatic testing – how to interpret and manage germline implications**  **Dr Jamshid Khorashad** |
| 09:55 to 10:40 | **Consensus statements and polling**  **Chaired by Dr Katie Snape** |
| 10:40 to 11:00 Break | |
| 11:00 to 11:35 | **Myeloproliferative neoplasms**  **What and how to report relating to phenotype/potential germline consequences for genes such as MPL, CSF3R, SH2B3, JAK2?**  **Dr Angela Hamblin, Dr Anna Godfrey, Dr Adam Mead** |
| 11:35 to 12:35 | **Consensus statements and polling**  **Chaired by Dr Angela Hamblin** |
| 12:35 to 12:45 | **End of meeting round up**  **Bev Speight** |
